# Formulæ for the Antiseptic Treatment of Boils and Carbuncles

**Published:** 1894-12

**Authors:** E. Gourine


### FORMULÆ FOR THE ANTISEPTIC TREATMENT OF BOILS AND CARBUNCLES.—DR. E. GOURINE.

℞. Chloroform......... ℥j.

Essence of cloves .. M75-ʒv.

Creosote............ Mxv-xxx.

Camphorated oil.... ℥ij.

Mix.—For external use only.

℞. Salicylate of mercury gr. ij-v.

Salicylic acid....... gr. xv-xxx.

Rectified spirits.... ℥ss-j.

Distilled water ..... ad. ℥iv.

Mix.—For external use only.

The affected part is covered with compresses saturated with either of the above antiseptic mixtures. At the same time, one or the other of the following solutions is injected into the boil or carbuncle:

℞. Carbolic acid ......... gr. j-iij.

Salicylate of sodium }

Borax .............. } aa gr. xv.

Glycerine.......... Mxxx.

Chloroform water..... ʒij

F. S. A.

℞. Iodoform .......... gr. ʌ-viij.

Salol................ gr. viij xv.

Carbolic acid ...... gr. iss.

Ether ............... ʒss-j.

Rectified spirits.... ʒiss-ij.

F. S. A

This treatment is said to give excellent results and to do away with the necessity for a more radical surgical intervention even in cases of carbuncle.—*Medical Week.—Ex.*

---

AMONGST the remedies employed in the treatment of scrofulous glands, the physician who has had any experience with the Elixir Six Iodides (W-G's) accepts the fact that it is the most effective combination he possesses.

